# Recent advances in the synthesis of biologically and pharmaceutically active quinoline and its analogues: a review

**DOI:** 10.1039/d0ra03763j

**Published:** 2020-06-02

**Authors:** Abdanne Weyesa, Endale Mulugeta

**Affiliations:** Department of Applied Chemistry, School of Applied Natural Science, Adama Science and Technology University P. O. Box: 1888 Adama Ethiopia endale.mulugeta@astu.edu.et

## Abstract

Recently, quinoline has become an essential heterocyclic compound due to its versatile applications in the fields of industrial and synthetic organic chemistry. It is a vital scaffold for leads in drug discovery and plays a major role in the field of medicinal chemistry. Nowadays there are plenty of articles reporting syntheses of the main scaffold and its functionalization for biological and pharmaceutical activities. So far, a wide range of synthesis protocols have been reported in the literature for the construction of this scaffold. For example, Gould–Jacob, Friedländer, Pfitzinger, Skraup, Doebner–von Miller and Conrad–Limpach are well-known classical synthesis protocols used up to now for the construction of the principal quinoline scaffold. Transition metal catalysed reactions, metal-free ionic liquid mediated reactions, ultrasound irradiation reactions and green reaction protocols are also useful for the construction and functionalization of this compound. The main part of this review focuses on and highlights the above-mentioned synthesis procedures and findings to tackle the drawbacks of the syntheses and side effects on the environment. Furthermore, various selected quinolines and derivatives with potential biological and pharmaceutical activities will be presented.

## Abstract 2

Recently, quinoline has become an essential heterocyclic compound due to its versatile applications in the fields of industrial and synthetic organic chemistry.

## Graphical abstract

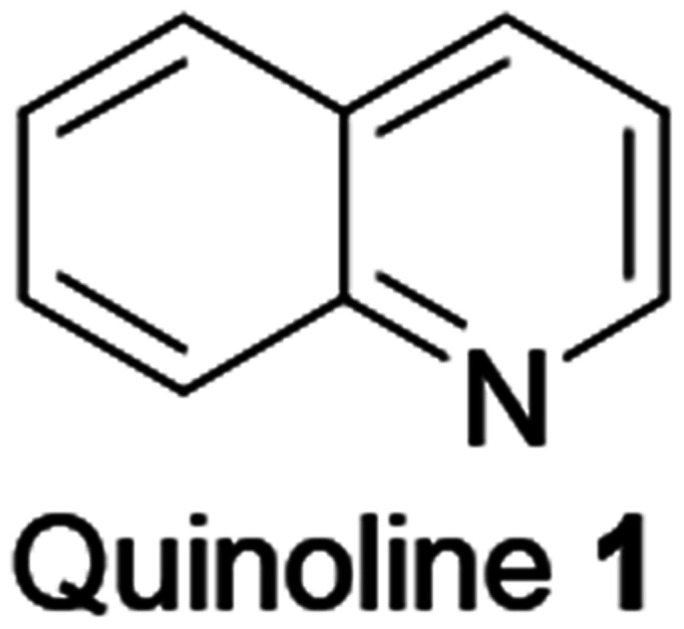

## 1. Introduction

**Fig. 1:**
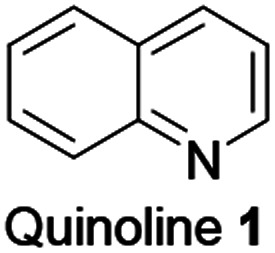
Chemical structure of quinoline.

Quinoline is the most ubiquitous heterocyclic aromatic compound with a potential for industrial and medicinal applications. 1-Azanapthalene and benzo[*b*]pyridine are used as alternative names for quinoline (Fig. 1).

It has a characteristic double-ring structure containing a benzene ring fused with a pyridine moiety, with the molecular formula C_9_H_7_N.^1^ Quinoline is an essential segment of both natural and synthetic compounds. In particular, the pyranoquinoline ring system has gained considerable attention as it is a core structure, constituting the basic skeleton of a number of alkaloids.^2^ Generally quinoline is present in pharmacologically active natural products and in synthetic products. This compound is used mainly as a central template for the synthesis of various drugs. Quinoline is a weak tertiary base and can form salts with acids. It exhibits similar reactions to pyridine and benzene and can also participate in both electrophilic and nucleophilic substitution reactions. It is nontoxic to humans.^3^ For the construction and functionalization of this noble compound and its derivatives, an enormous number of synthesis techniques have been reported, among which conventional or classical methods, transition metal free catalysed methods, ultrasound irradiation reactions and greener chemical processes have been well explored. Most of the time scholars explore classical reaction methodologies, such as Gould–Jacobs,^4^ Friedländer,^5^ Pfitzinger,^6^ Skraup,^7^ Doebner–von Miller,^8^ and Conrad–Limpach,^9^ and modify them with eco-friendly transition metal mediated, ultrasound irradiation reactions or greener protocols.^10,11^

Currently, studies revealing numerous natural products and synthetic derivatives incorporating a quinoline scaffold have attracted scholars' attention because they exhibit a broad range of biological and pharmaceutical activities.

**Fig. 2:**
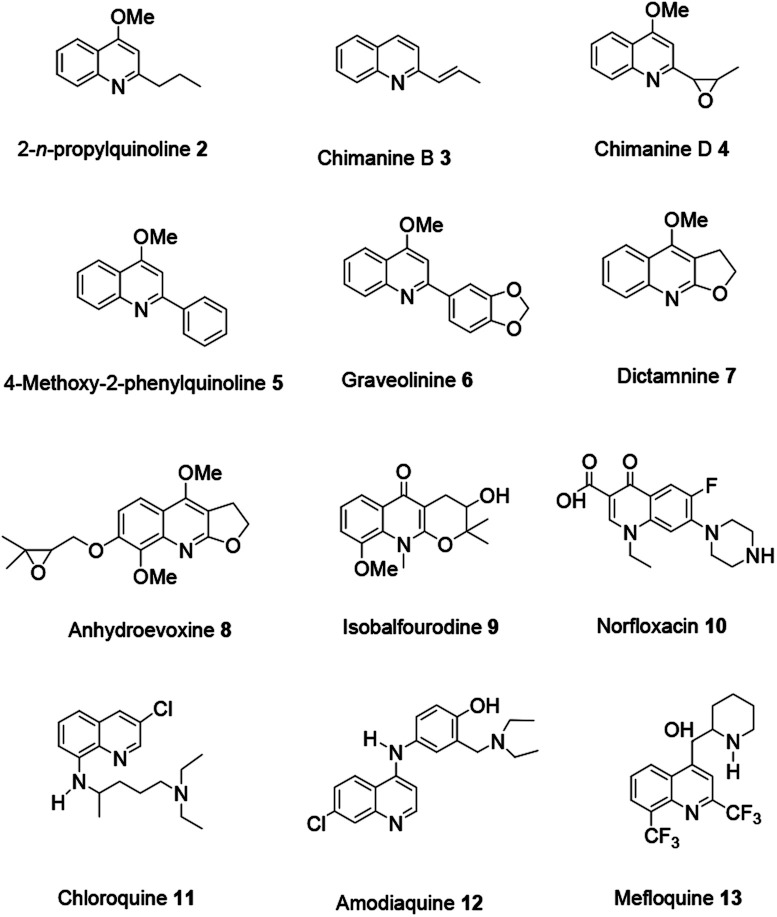
Structures of bioactive compounds from natural sources.

For example antibacterial, antioxidant, anticancer, anti-inflammatory, antimalarial, antifungal and antileishmanial activities have been well studied.^12^ Shang and co-workers reported a comprehensive review on alkaloids with a quinoline moiety as a core scaffold, isolated from compounds from natural sources and showing bioactivity potential.^13^ The review comprehensively organized into two focal sections. First various synthesis strategies will be addressed to highlight the original reaction procedures and modifications in the recent literature related to all the synthetic strategies accordingly. Then novel pharmaceutically and biologically active quinolines will be explored (Fig. 2).

## 2. Synthesis of quinoline and its derivatives

So far scholars have explored copious synthesis protocols to construct and functionalize the quinoline scaffold. Here, we focus on a review of the classical synthesis procedures named Gould–Jacob, Friedländer, Pfitzinger, Skraup, Doebner–von Miller, and Conrad–Limpach reactions, transition metal catalysed reactions, transition metal free ionic liquid mediated reactions, ultrasound irradiation reactions, and greener chemical processes for the synthesis of quinoline and its analogues will be discussed briefly.

### 2.1. Gould–Jacob quinoline synthesis

**Scheme 1:**
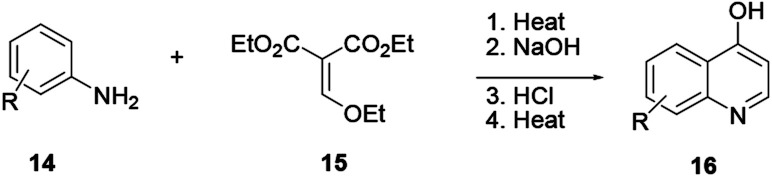
General scheme of the Gould–Jacobs quinoline synthesis.

Various quinoline scaffolds with substituents at carbon-4 can be prepared through cascade reactions known as the Gould–Jacob cyclization reaction.^4^ In this procedure 4-hydroxyquinoline 16 is prepared from aniline (14) and diethyl ethoxymethylenemalonate (15) involving series of reactions to provide quinoline 16 (Scheme 1).

This protocol helps in the preparation of various commercially available drugs based on a quinoline scaffold as the core skeleton. For instance, the preparation of the well-known nonsteroidal anti-inflammatory drugs floctafenine and glafenine relies on this synthesis methodology.^4^

### 2.2. Friedländer quinoline synthesis

**Scheme 2:**
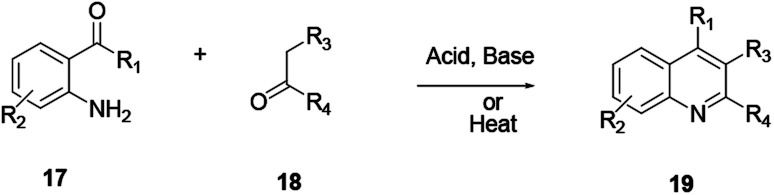
General reaction scheme of Friedländer quinoline synthesis.

In this procedure *ortho*-substitution of aniline 17 and aldehyde or ketone 18 with a reactive α-methylene group *via* condensation followed by cyclodehydration reactions affords compound 19 (Scheme 2). In this reaction procedure, regioselectivity is a challenging issue when unsymmetrical ketones are used.^5^ The reaction is well catalysed using a base or acid, such as a Brønsted acid or a Lewis acid, and ionic liquids can also activate the reaction well. Furthermore, it can proceed smoothly without a catalyst by heating the mixture. The merit of this reaction procedure is the scope of substrates of various functional groups that are well tolerated on both arylamine and ketone moieties.

### 2.3. Modified Friedländer quinoline synthesis

**Scheme 3:**
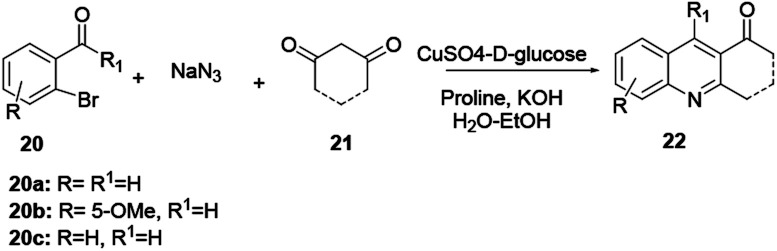
General reaction scheme of modified Friedländer quinoline synthesis.

Anand and co-workers operationally developed a simple, highly efficient, practical and convenient one-pot method for the synthesis of a broad scope of quinoline derivatives.^6^ Here, by using 2-bromobenzaldehyde (20), acyclic or cyclic 1,3-diketone (21) and sodium azide in a three-component reaction protocol in the presence of an air-stable, eco-efficient and inexpensive catalyst, quinoline 22 is prepared in good yields. In this reaction copper salt-d-glucose helps to generate Cu(i) species *in situ* through reduction in aqueous ethanol as a green solvent, and proline is used as a ligand and proton source to synthesize the target compound (Scheme 3). This protocol follows an Ullmann-type coupling reaction where nucleophilic substitution of Br from 2-bromobenzaldehyde (20) with sodium azide gives an azido complex intermediate. The azido–Cu complex is subjected to reductive elimination followed by dehydrative cyclocondensation, providing the desired quinoline 22.

The authors claim that this method worked nicely with both electron-donating and electron-withdrawing substituent groups at the *ortho*-, *meta*-, and *para*-positions of the phenyl ring.^6^

### 2.4. Pfitzinger quinoline synthesis

**Scheme 4:**
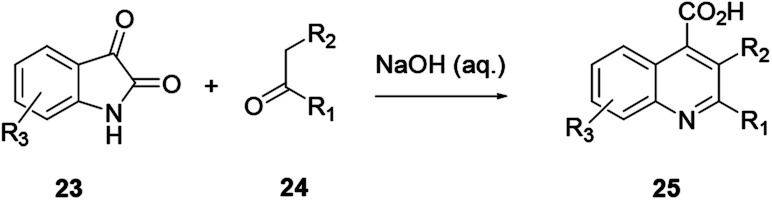
General reaction scheme of the Pfitzinger quinoline synthesis.

**Scheme 5:**
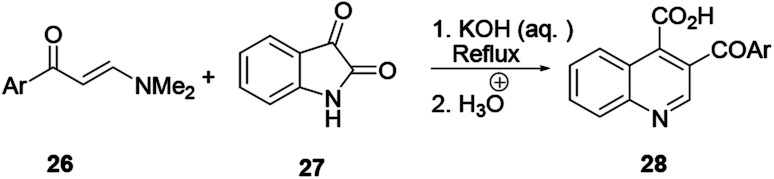
Synthesis of quinoline derivative 28 through the Pfitzinger reaction.

This procedure is also known as the Pfitzinger–Borsche reaction. Here isatin 23 reacting with α-methylene carbonyl compound 24 in the presence of a base in ethanol provides substituted quinoline derivative 25 (Scheme 4).^6^ In this reaction protocol, isatic acid is formed from isatin 23 and condensed with α-methylene carbonyl compound 24 in the presence of a strong base. Subsequent decarboxylation affords quinoline 25. This procedure is an extension of the Friedländer quinoline synthesis protocol. The reaction basically depends on the more stable isatin varieties instead of the *ortho*-aminoaryl moieties that are the basic starting materials for the preparation of quinoline *via* the former reaction protocol.^7^ Elghamry and co-workers used a similar reaction protocol with minor modifications in a one-step and one-pot method to synthesize quinoline-4-carboxylic acids 28 in water (Scheme 5). Isatin (27) was refluxed with enaminone 26 in the presence of an aqueous solution of KOH or NaOH, and subsequent acidification using dilute HCl to prepare quinoline-4-carboxylic acid (28) in good to excellent yields.^7^ The authors claimed that using enaminone as a replacement for 1,3-dicarbinols improves the yield and practicality of the reaction.

### 2.5. Skraup/Doebner–von Miller quinoline synthesis

**Scheme 6:**
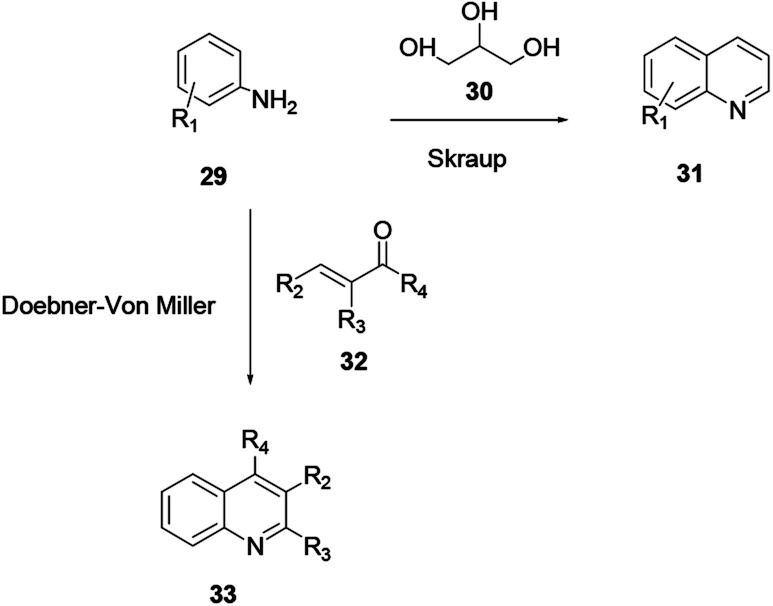
General reaction scheme of Skraup/Doebner–von Miller quinoline synthesis.

A synthesis of quinoline *via* aniline and glycerine in the presence of a strong acid and an oxidant under reflux was revealed by Skraup and co-workers.^8^ Here, a crotonaldehyde intermediate is generated *in situ* from glycerol 30. Subsequently aniline 29 is added to the reaction under heating to provide quinoline 31. The reaction of substituted acrolein 32 with aniline 29 in the presence of an oxidant to provide quinoline 33 is known as the Doebner–von Miller protocol (Scheme 6).

**Scheme 7:**
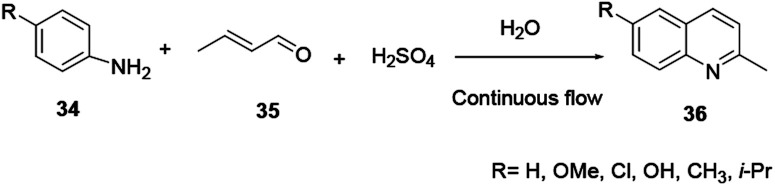
General reaction scheme of Doebner–von Miller quinoline synthesis.

The fundamental drawbacks of the Skraup and Doebner–von Miller syntheses are that both turn out to be violently exothermic during the progress of the reaction, and the variety of oxidants and the highly acidic medium required make isolation of the desired product tedious. Regioselectivity is also a concern when *meta* or 3,4-disubstituted anilines are used.^9^ 2-methylquinoline and its derivatives have shown substantial biological activities. However, there are different techniques for the synthesis of 2-methylquinoline, and Doebner–von Miller is the best. Yalgin and co-workers report the synthesis of 2-methylquinoline 36 using a modified Doebner–von Miller reaction protocol in the presence of a strong acid in a flow reactor with aniline and acrolein.^15^ 2-Methylquinoline derivative **36** synthesized using a continuous flow in water through Doebner–Miller reaction procedure. This method is a rapid and green route for the synthesis of quinoline derivatives to provide a good to excellent yields (Scheme 7).^14^

### 2.6. Combes/Conrad–Limpach quinoline synthesis

**Scheme 8:**
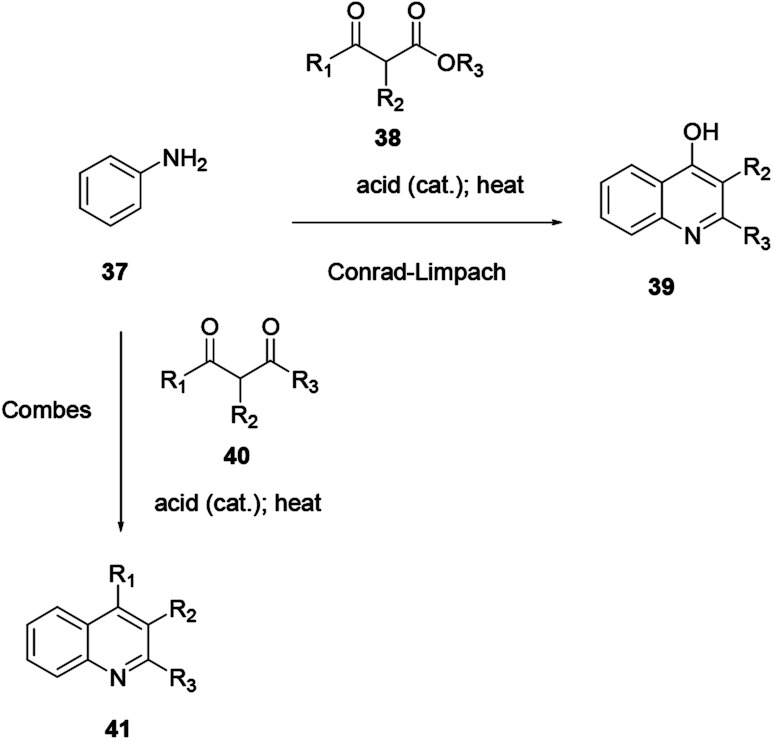
General reaction scheme of the Combes/Conrad–Limpach quinoline synthesis.

The Combes/Conrad–Limpach reaction is the condensation of a primary aryl amine with a 1,3-diketone or β-keto-aldehyde or 1,3-dialdehyde, giving an enamine intermediate which is further heated in a strong acid, and later cyclodehydration to afford quinoline. Here, the condensation of primary aryl amine 37 with β-diketone 40 in acid catalysis, followed by ring closure of a Schiff base, affords quinoline 41 (Scheme 8).^10^

Using the same reaction procedure the Conrad–Limpach scheme can be used to prepare various quinoline derivatives using β-ketoester 38 in place of β-diketone 40. Both procedures follow the condensation of arylamine with β-ketoester or β-diketone by a further cyclodehydration reaction and heating in strong acid to prepare the target compounds.

### 2.7. Ultrasound irradiation reactions

**Scheme 9:**
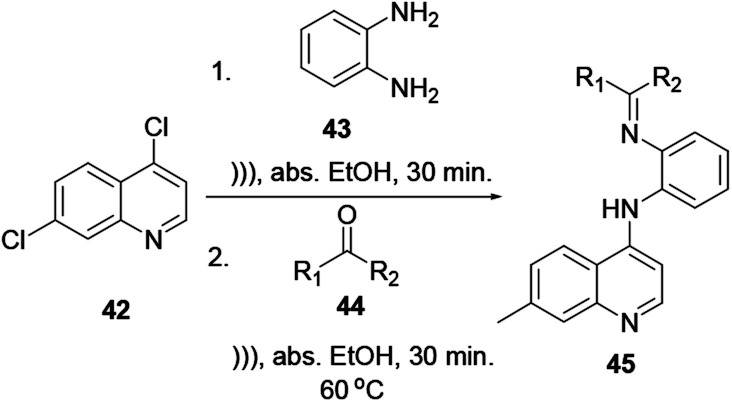
Synthesis of a quinoline derivative using ultrasound irradiation.

The reaction time, product yields and qualities of this reaction procedure are better than those from the above-mentioned quinoline synthesis protocols. It is one of the greener quinoline synthesis protocols. Using an ultrasound irradiation reaction procedure *via* two sequential reactions, S_N_2 followed by a condensation reaction, quinoline 45 can be synthesized in good yield (Scheme 9).^12^

This procedure has the advantages of a short reaction time and easy isolation of the product and provides good-to-excellent yields.^16^ Quinoline 45 can be easily functionalized at the 4-chloro position with *o*-phenylenediamine 43, and further reacted with unsymmetrical ketone 44*via* a green chemistry protocol to provide quinoline derivative 45.

**Scheme 10:**
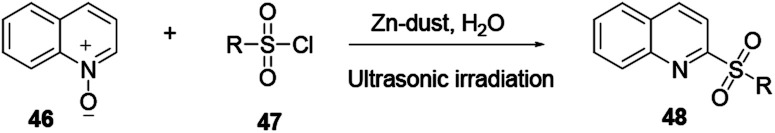
Synthesis of a quinoline derivative using a non-metal catalyst.

Xie and co-workers report C2-sulfonation of *N*-oxide quinoline (46) using sulfonyl chloride 47 in the presence of Zn-dust, with water as a solvent *via* an ultrasound-assisted protocol, providing quinoline 48 in moderate yield.^17^ Here, sulfonyl chloride 47 is first reduced using Zn-dust. The generated intermediate is coordinated with *N*-oxide quinoline 46. Finally, the target compound 48 is synthesized through an intramolecular nucleophilic addition reaction (Scheme 10).

### 2.8. Transition metal free quinoline synthesis

**Scheme 11:**
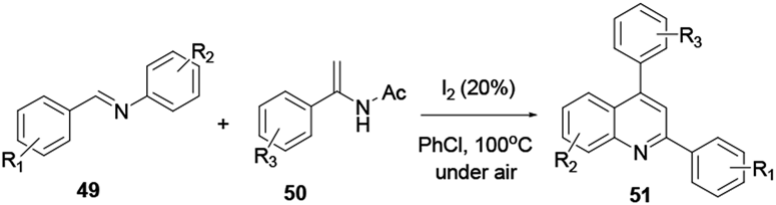
Synthesis of a quinoline derivative using a non-metal catalyst.

Various scholars report the synthesis of quinoline and its derivatives *via* metal-free mediated reaction protocols, such as ionic liquid, simple acid or base catalyst, using molecular iodine and catalyst-free reactions. The above-mentioned procedures are considered to be green chemical processes. Conducting reactions using the above strategies can achieve a high level of atom efficiency. The reaction media are recyclable, they provide high yields, have a short reaction time, are practical to operate under mild reaction conditions, conduct the reduction in a number of steps, decrease waste and are eco-friendly.^18^ Here, two amine derivatives, enamide 49 and imine 50, react in the presence of iodine in air to afford quinoline 51 (Scheme 11). In order to improve the reaction efficiency, various types of catalysts were explored. Among all these catalysts, iodine exhibited the highest yields. Moreover, the reaction has attracted a great deal of attention with its benefits of low toxicity, ability to operate under mild reaction conditions, low-cost starting materials and broad scope of substrates.

**Scheme 12:**
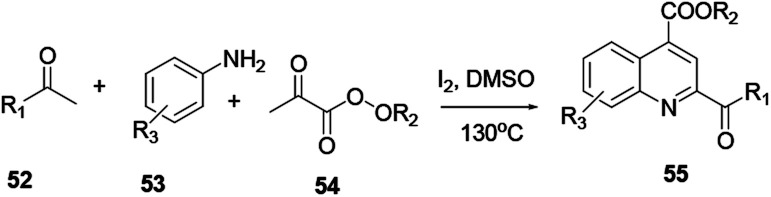
Three-component iodine-catalysed quinoline synthesis.

**Scheme 13:**
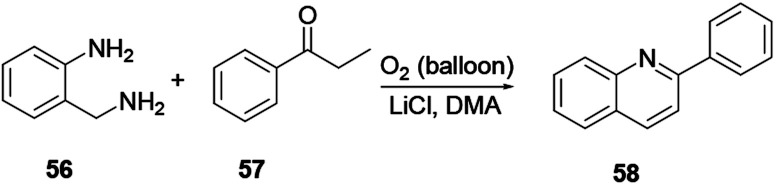
Metal-free, aerobic quinoline synthesis.

The three-component reaction of methyl ketone 52, arylamine 53, and α-ketoester 54 in the presence of iodine and a catalytic amount of hydroiodic acid provides quinoline 55. In this reaction the HI co-product acts as a promoter with good functional compatibility (Scheme 12).^19^ Under transition metal free reaction conditions oxygen acts as an oxidant, where 2-(aminomethyl)-aniline 56 reacts with aromatic ketone 57 to provide the corresponding quinoline 58 with excellent yield (Scheme 13). Wu and co-workers reported that the reaction procedure for the synthesis of quinoline can be achieved under aerobic reaction conditions without the presence of a catalyst, with lithium chloride salt additive in dimethylacetamide solvent.^20^ Following this reaction procedure ketones bearing strong electron-withdrawing or electron-donating groups could react smoothly and in an eco-friendly way to provide the desired product.

**Scheme 14:**
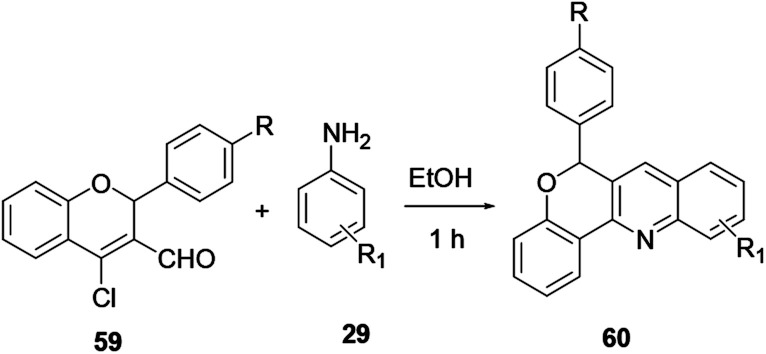
Synthesis of quinoline derivative 60.

Carbaldehyde 59 reacted with substituted aniline 29 without a catalyst to provide quinoline 60 in excellent yield (Scheme 14).^21^ The reaction is performed at room temperature, with a short reaction time, providing excellent yield with no catalyst required (Scheme 14).

**Scheme 15:**
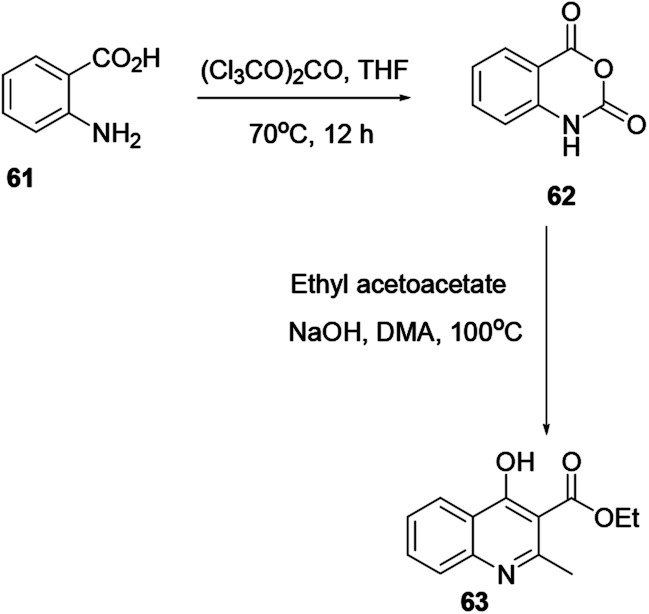
Synthesis of quinoline derivative 63.

Through a convenient two consecutive steps ethyl-4-hydroxy-2-methylquinoline-3-carboxylate (63) was synthesized. Using solid triphosgene in THF, commercially available 2-aminobenzoic acid 61 converted to isatoic anhydride 62, reacted with the sodium enolate of ethyl acetoacetate in warm DMA, to afford substituted quinoline 63 (Scheme 15).^22^

**Scheme 16:**
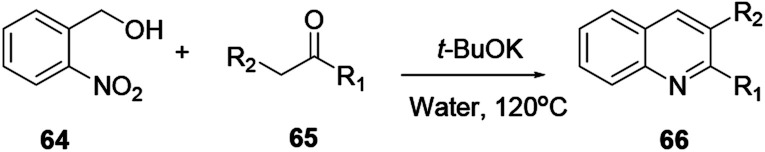
Synthesis of quinoline 66 in water.

Zhu and co-workers reported an environmentally friendly reaction methodology to prepare quinoline 66.^23^ In this reaction procedure the cheap and commercially available material 2-nitrobenzyl alcohol (64) reacted with ketone 65*via* an intramolecular redox process to afford synthetically important quinoline 66 in moderate yields (Scheme 16).

**Scheme 17:**
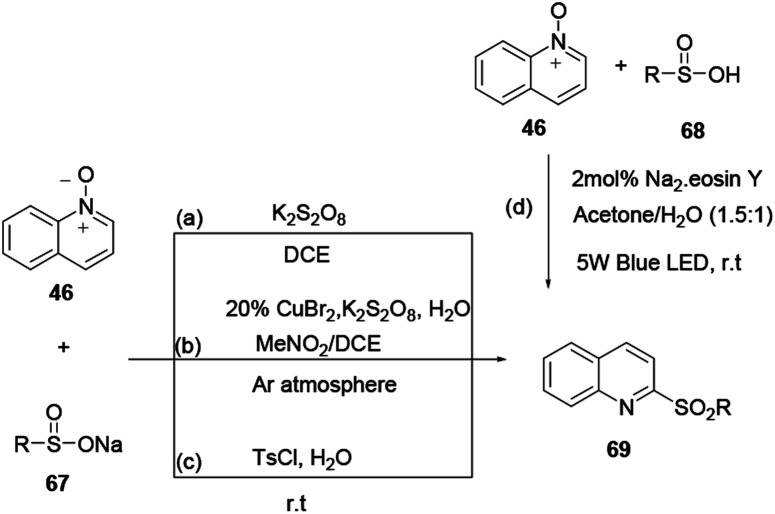
Synthesis of quinoline 69 through various reaction conditions.

2-Sulfonylquinoline 69 was prepared smoothly without a base or organic solvent from quinoline *N*-oxide 46 and sodium sulfinate 67 using a metal-free and dual radical coupling reaction protocol devised by Xie and co-workers.^24^ Sodium sulfinate is well-known for use as a sulfonating reagent in the following reactions, because it is stable to moisture and is safe and easy to handle. Additionally, Han and co-workers report the synthesis of 2-sulfonylquinoline 69 using quinoline *N*-oxide with sodium sulfinate as starting materials in the presence of K_2_S_2_O_8_ as an oxidant under an inert atmosphere in a mixed solvent system.^25^ Peng and co-workers amended previously reported protocols and reported the synthesis of 2-sulfonylquinoline 69 from quinoline *N*-oxide and sodium sulfinate *via* an easy, metal-free, oxidant-free and solvent-free method under mild reaction conditions using TsCl in water.^26^ Furthermore, 2-sulfonylquinoline 69 was synthesized from quinoline *N*-oxide and sulfonic acid 68 using organic dye as a catalyst (Scheme 17).^27^

The reaction was performed with a greener process protocol, and in ambient air to provide good to excellent yields.

### 2.9. Transition metal mediated protocols

**Scheme 18:**
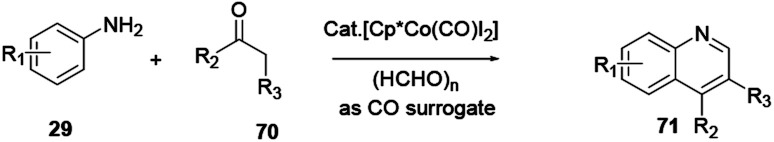
Cobalt-catalysed synthesis of quinoline derivatives.

Xu and co-workers reported the one-pot synthesis of substituted quinoline 71 with a broad scope of substrates using a transition Co(iii) metal mediated reaction protocol.^28^ Co(iii) catalysis *via* a cascade of reactions, C–H activation, carbonylation and cyclization of aniline 29 and ketone 70 with paraformaldehyde provide various useful quinoline derivatives (Scheme 18). Benefits of the procedure are the broad scope of substrates with tolerance to various functional groups and the affordance of good to excellent yields. Moreover, it releases water and hydrogen gas as by-products.

**Scheme 19:**
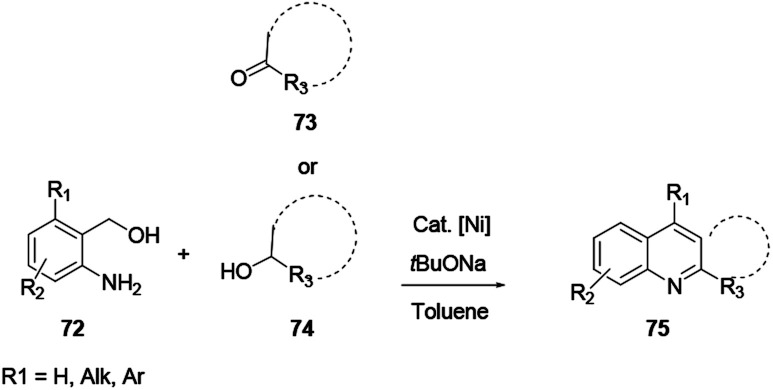
Nickel-catalysed synthesis of polysubstituted quinolines.

**Scheme 20:**
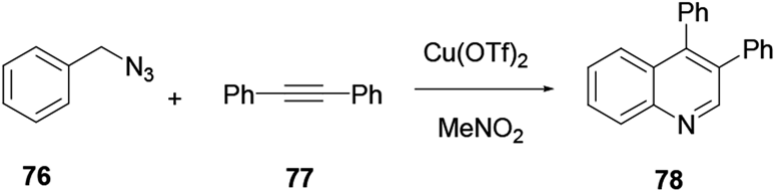
Copper(ii) triflate mediated synthesis of a quinoline derivative.

**Scheme 21:**
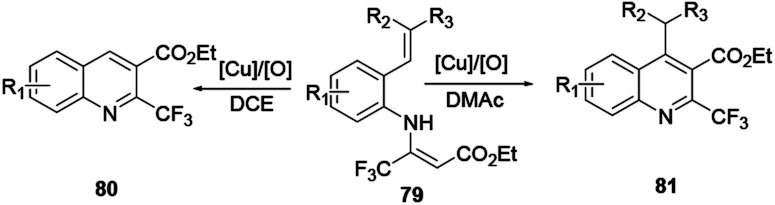
Synthesis of 2-trifluoromethylquinoline.

Das and co-workers reported the synthesis of various polysubstituted quinolines 75 from commercially available α-aminoaryl alcohol 72 and acyclic/cyclic ketones 73 or secondary alcohol 74*via* a sequential dehydrogenation and condensation reaction process (Scheme 19).^29^ The advantage of conducting reactions *via* this method is that it uses a simple-to-handle and inexpensive nickel(ii) catalyst. Luo and co-workers reported 6-chloro-2-dimethyl-4-phenylquinoline, an antiparasitic agent, and 3,4-diphenylquinoline-2(1*H*)-one, a p38αMAP kinase inhibitor, as biologically active quinoline derivatives. The reaction proceeds through a single-step process and under neutral reaction conditions using a copper triflate catalyst in nitromethane.^30^ In contrast to an acid-catalysed two-step [4 + 2] cycloaddition and oxidation reaction, it allows a single-step reaction to provide quinoline in high yields and excellent regioselectivity (Scheme 20). *N*-(2-alkenylaryl)enamine 79 is a strategic precursor for the synthesis of quinoline 80 or 81 using a one-pot copper-catalysed aerobic oxidative cyclization reaction (Scheme 21). Chen and co-workers reported a novel and efficient procedure for the synthesis of 2-trifluoromethylquinolines 80 and 81 using copper-chloride salt under aerobic reaction conditions with various functional group tolerances.^31^

**Scheme 22:**
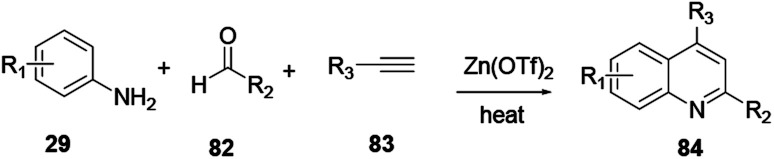
Zinc triflate mediated synthesis of quinoline derivatives.

2,3-Disubstituted quinoline 84 was synthesized through multicomponent coupling reactions of alkyne 83, amine 29 and aldehyde 82 using a zinc(ii) triflate salt catalyst (Scheme 22).^32^ The merits of this procedure are that reactions can proceed without the presence of ligands or co-catalysts, under solvent-free and inert reaction conditions. Bao and co-workers recently reported a simple and efficient synthetic protocol for the construction of sulfonylated quinoline in water *via* a zinc powder mediated coupling reaction.^33^

**Scheme 23:**
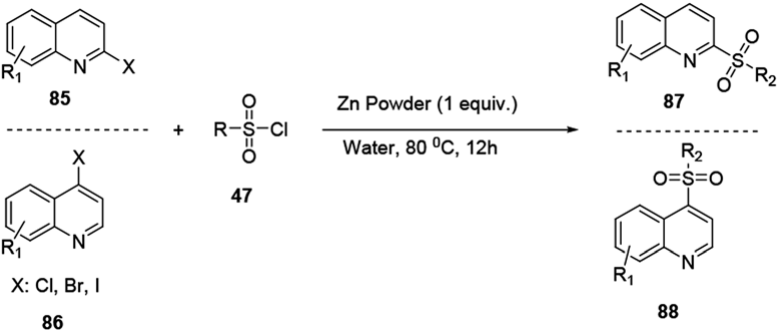
Zinc powder mediated synthesis of sulfonylated quinoline derivatives.

Haloquinolines 85 and 86 reacted with sulfonyl chloride in water in the presence of cheap metal zinc powder to afford sulfonylated quinolines 87 and 88, respectively (Scheme 23).

**Scheme 24:**
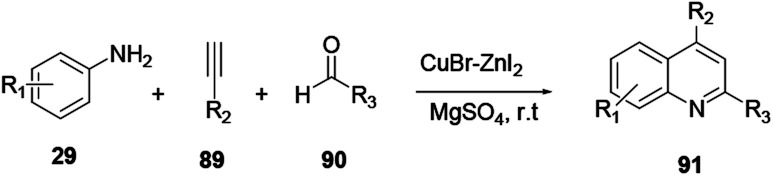
Copper–zinc combo-catalysed synthesis of quinoline derivatives.

Mondal and co-workers reported a neat Zn^II^/Cu^I^ catalyzed reaction procedure to synthesise quinoline 91 through the uncommon sp^2^ C–H activation of three-component protocols.^34^ Aniline 29, alkynes 89 and aldehydes 90 react *via* a cascade cyclization reaction to provide quinoline 91 (Scheme 24).

**Scheme 25:**
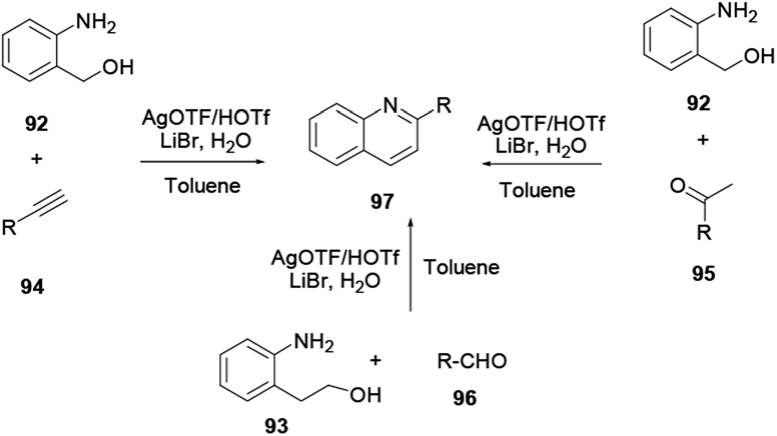
Silver triflate catalysed synthesis of quinoline derivatives.

2-Substituted quinoline 97 is prepared from either 2-aminobenzyl alcohol and alkyne/ketone or 2-aminophenethyl alcohol and aldehyde using an AgOTf catalyst.^35^ Synthetically important heterocyclic anchored quinolines, such as furan, pyrrole and thiophene, can be synthesized *via* a facile and economic procedure using a silver triflate catalyst in toluene solvent with commercially available precursors and additives, as reported by Xu and co-workers (Scheme 25). Afterwards, the silver triflate catalysed one-step synthesis of quinoline with a broad scope of substrates was reported by Xu and co-workers.^36^

**Scheme 26:**
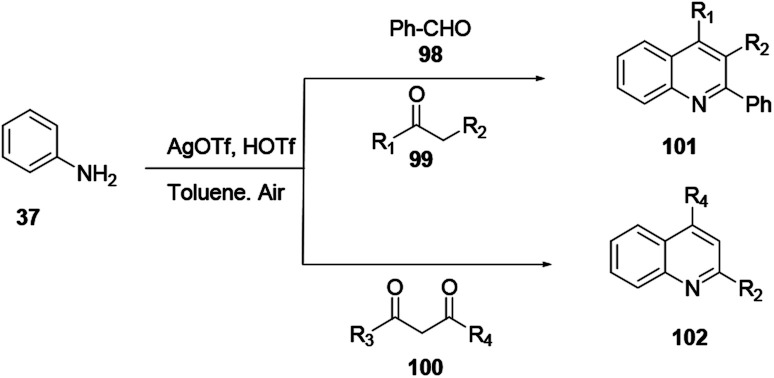
Silver triflate mediated synthesis of polysubstituted quinolines.

Multicomponent reactions proceeding using aniline 37, aldehyde 98, and ketones 99 or 100 reacting in the presence of a silver triflate catalyst provide quinolines 101 or 102 (Scheme 26).

**Scheme 27:**
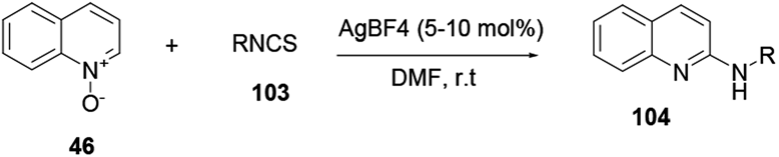
Silver-catalysed amination of quinoline *N*-oxides.

Xie and co-workers reported an effective and suitable protocol for the synthesis of 2-aminoquinoline 104 using an AgBF_4_ catalyst in DMF solvent.^37^ The reaction proceeds using isothiocyanate 103 under basic and oxidant-free mild reaction conditions to provide good to excellent yields (Scheme 27).

**Scheme 28:**
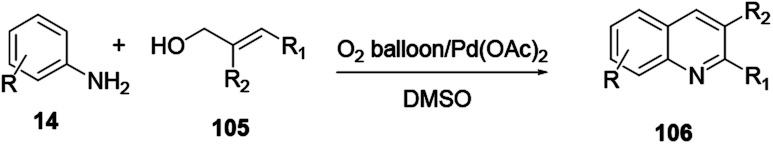
Palladium-catalysed synthesis of quinoline derivatives.

The reaction of commercially available starting materials aniline 14 and aryl allyl alcohol 105 using a palladium acetate catalyst in DMSO solvent *via* an oxidative cyclization reaction provides quinoline 106 (Scheme 28).^38^ It does not need to employ any acid, base or additive to promote the reaction. The procedure works with a broad scope of substrates and strong electron-withdrawing substituted materials to furnish moderate to satisfactory yields.

## 3. Bioactivities of quinolines

Both natural products and synthetic compounds which are anchored with a quinoline scaffold have certainly exhibited a broad range of biological or pharmaceutical activities.^39,40^ Among them are antibacterial,^41,42^ antioxidant,^43^ anticancer,^44^ anti-inflammatory,^45,46^ antimalarial,^47,48^ and antifungal activities.^49^

### 3.1. Antibacterial activity

**Fig. 3:**
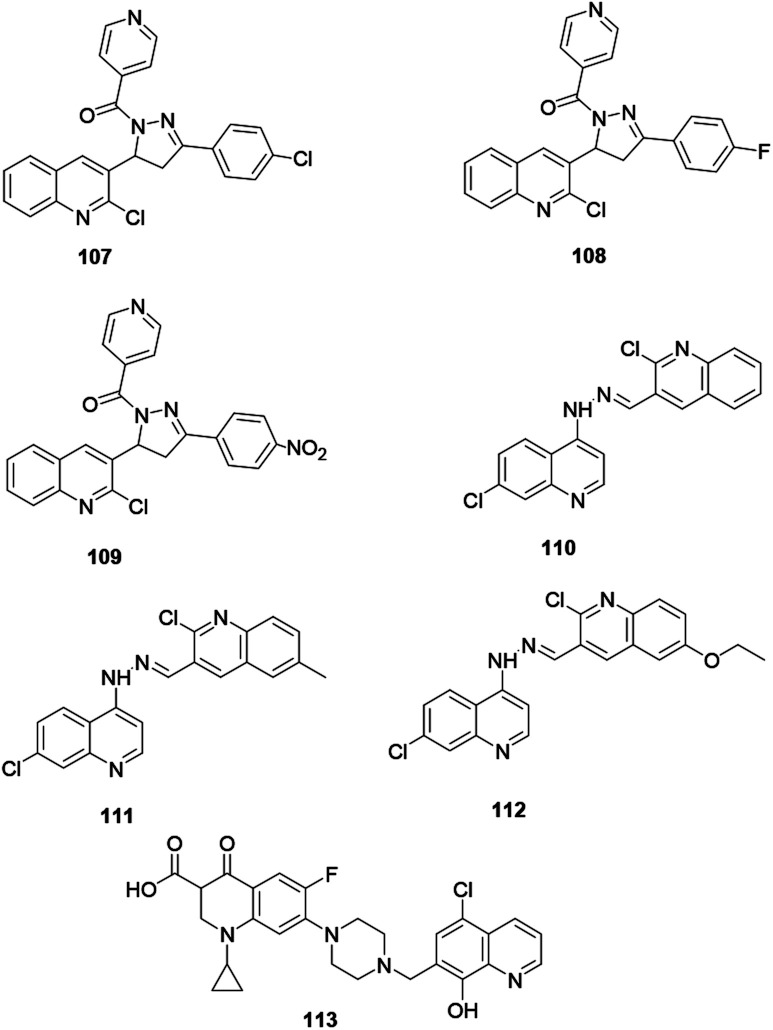
Chemical structures of antibacterially active quinoline derivatives.

Desai and co-workers reported the synthesis of quinoline derivatives 107, 108 and 109 exhibiting the most powerful antimicrobial activities (Fig. 3).^50^ The synthesized compounds were screened for their potential antibacterial activity on *Staphylococcus aureus*, *Streptococcus pyogenes*, *Escherichia coli*, and *Pseudomonas aeruginosa* using ampicillin as a standard drug. The recorded results revealed that these compounds have good bacterial activity with minimum inhibition concentrations of 12.5 μg ml^−1^ and 50 μg ml^−1^. Furthermore, the authors claim that the potential activity of these compounds is directly associated with the substituent effect on the ring. These three bioactive quinoline derivatives are linked using hydrazone, as reported by Le and co-workers.^51^ Quinoline derivatives with a hydrazone linker 110, 111 and 112 showed good growth inhibition of targeted bacteria.

Furthermore, Fu and co-workers synthesized hybridized quinoline derivative 113 with a piperazine moiety linker and reported that it showed broad-spectrum antibacterial activities on selected bacteria with MIC values of 0.125–8 μg ml^−1^.^52^

### 3.2. Antioxidant activity

**Fig. 4:**
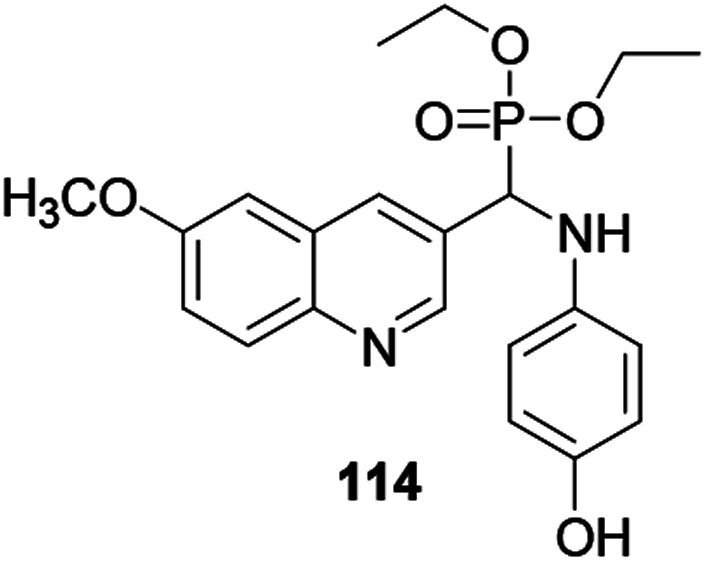
Chemical structure of an antioxidant active quinoline derivative.

Bazine and co-workers report that quinoline derivative 114 anchored with an α-aminophosphate revealed effective antioxidant activity when compared with standard DPPH (Fig. 4).^53^ The authors confirmed that the bioactivity was further modified through the introduction of a phenol ring as a substituent to the quinoline scaffold.

### 3.3. Anticancer activity

**Fig. 5:**
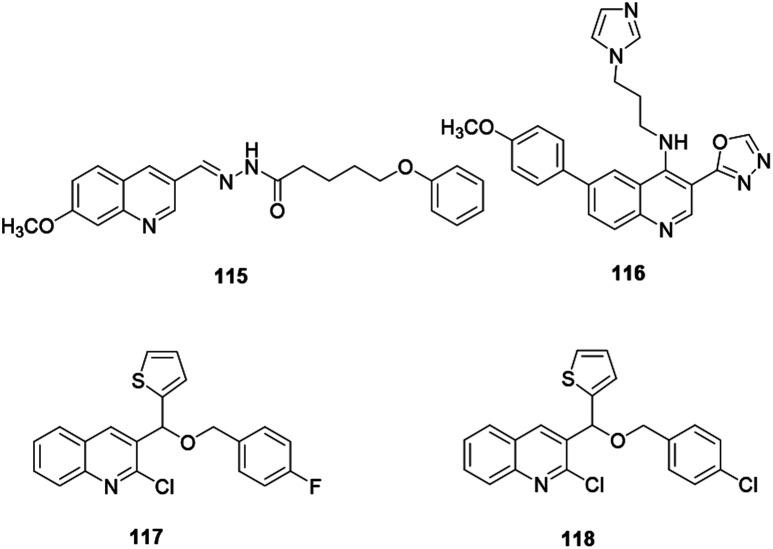
Chemical structures of anticancer active quinoline derivatives.

Bingul and co-workers synthesized and reported quinoline derivative 115 for anticancer activity against neuroblastoma cells (Fig. 5). The authors revealed that compound 115 showed reasonable anticancer activities against SH-SY5Y and Kelly neuroblastoma cell lines and decreased the viability of neurocancer cells with substantial selectivity over normal cells.^54^ Compounds 117 and 118 are quinoline derivatives anchored with a thiophene moiety, reported by Othman and co-workers.^55^ The above quinolines exhibited powerful anticancer activity against MCF-7 human cancer cells with IC_50_ values of 38.41 and 28.36 μM, respectively. Additionally, Kundu and co-workers reported that quinoline 116 with an imidazole and 1,3,4-oxadiazole exhibited the highest potency for human topoisomerase 1.^56^

### 3.4. Anti-inflammatory activity

**Fig. 6:**
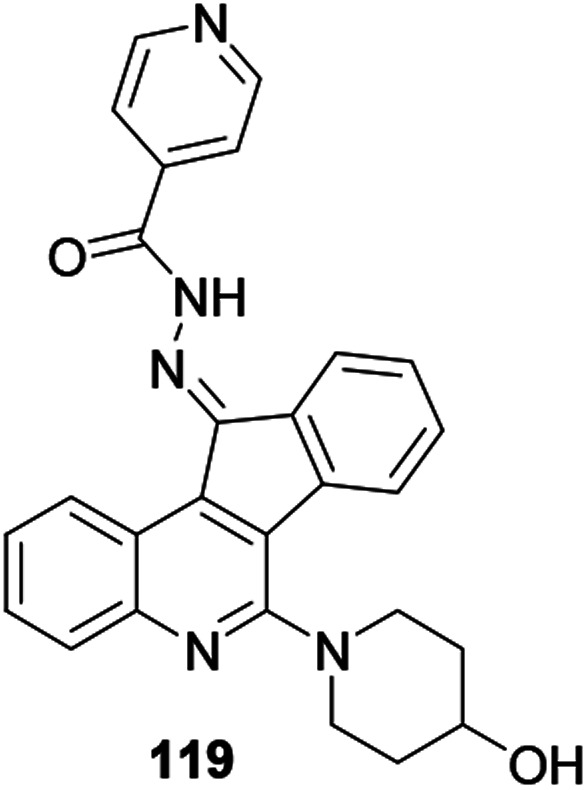
Chemical structure of anti-inflammatory active quinoline derivative.

Tseng and co-workers synthesized and reported indeno[1,2-*c*] quinoline derivatives 119 as a potent anti-TB agent, besides being a potent anti-inflammatory agent with low cytotoxicity (Fig. 6).^57^

### 3.5. Antileishmanial activity

**Fig. 7:**
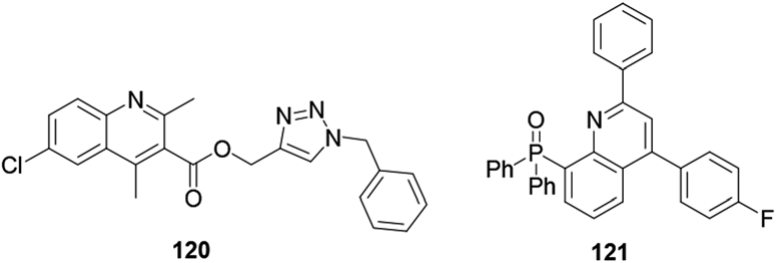
Chemical structure of antileishmanial active quinoline derivative.

Upadhyay and co-workers synthesized and reported that quinoline derivative 120 anchored with a triazole exhibited antileishmanial activity (Fig. 7).^58^ The authors revealed that having a chloro-substituent enhanced the activity of the synthesized compound.

Phosphorated quinoline 121 is a hybrid and potent antileishmanial agent.^59^ Phosphorus hybridized with quinoline is a promising basis for strong antileishmanial activity.

### 3.6. Antimalarial activity

Currently, scholars are exploring how to improve and enhance the antimalarial activity of compounds with a quinoline scaffold. These are mainly synthesized quinoline derivatives hybridized with commercially available and potentially recognized drugs.^60^

**Fig. 8:**
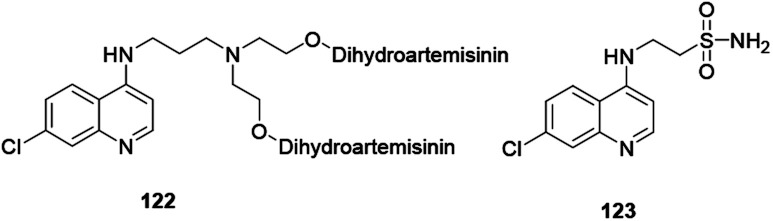
Chemical structures of hybridized quinolines.

Investigators claim that hybridization will result in benefits of cost-effectiveness and minimize the risk of drug–drug interaction. Lombard and co-workers reported quinoline hybridized with artemisinin drug and provided compound 122 (Fig. 8). The hybrid compound exhibited antimalarial activity, although not as much as dihydroartemisinin, but it showed excellent antiplasmodial activity.^61^ The antimalarially active quinoline–sulfonamide hybrid derivative 123 was synthesized and reported by Verma and co-workers.^62^ The authors revealed that the hybrid compound exhibited inhibition of the formation of hemozoin.

### 3.7. Antifungal activity

**Fig. 9:**
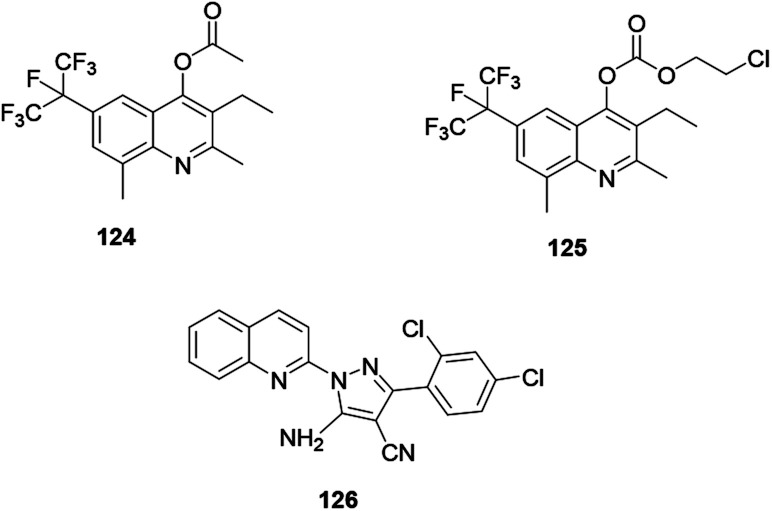
Chemical structures of antifungal active quinoline derivatives.

Antifungal active compounds 6-perfluoropropanyl quinoline 124 and 125 were synthesized and reported by Fang and co-workers (Fig. 9).^63^ The synthesized quinoline derivatives exhibited excellent antifungal activity against *Pyricularia oryzae*. El Shehry and co-workers synthesized and reported an antifungally active pyrazole–quinoline hybrid 126 (Fig. 9).^64^ The synthesized compound exhibited good antifungal activity against the target fungal species.

## 4. Conclusions

In conclusion, numerous quinoline derivatives play a big role in the progress of organic synthesis and applications to medicinal chemistry. Recently, researchers have synthesized hybrid quinoline scaffolds with compounds containing other heterocyclic compounds using different procedures. For example, through transitional metal catalysed or metal-free reactions, ultrasound irradiation, or multicomponent one-pot synthesis, modified named reactions can be instigated to synthesize various noble and effective quinoline derivatives. Generally, from the above-mentioned procedures metal-free, ionic liquid and ultrasound irradiation synthesis methodologies meet the requirements of ‘green chemistry’.

Until now the synthesis and modification of a quinoline scaffold and investigations to improve its biological and pharmaceutical activities have been continually developing.^65,66^ Nowadays researchers are focused not only on the synthesis of quinoline and its derivatives but also on developing and designing eco-friendly reaction procedures. Typical metal-free, solvent-free, aqueous media and ionic liquid catalysed reactions are green protocols.^67^ Quinoline and its derivatives have exhibited a potentially wide range of applications to treat various kinds of human infections, such as bacterial infections, cancer, malaria and fungal infections. The above-mentioned synthesis protocols based on green synthesis procedures are usually suggested for preparing this noble organic compound and its analogues.

## Conflicts of interest

Article content has no conflict of interest.

## Supplementary Material
